# Combined Effects of Repetitive Mild Traumatic Brain Injury and Alcohol Drinking on the Neuroinflammatory Cytokine Response and Cognitive Behavioral Outcomes

**DOI:** 10.3390/brainsci10110876

**Published:** 2020-11-19

**Authors:** Jessica Hoffman, Jin Yu, Cheryl Kirstein, Mark S. Kindy

**Affiliations:** 1 Department of Psychiatry, Bowles Center for Alcohol Studies, School of Medicine, University of North Carolina at Chapel Hill, Chapel Hill, NC 27599, USA; 2 Department of Pharmaceutical Sciences, College of Pharmacy, University of South Florida, Tampa, FL 33612, USA; jinyu@usf.edu; 3 Department of Psychology, College of Arts and Sciences, University of South Florida, Tampa, FL 33612, USA; kirstein@usf.edu; 4 James A. Haley VA Medical Center, Tampa, FL 33612, USA; 5 Shriners Hospital for Children, Tampa, FL 33612, USA

**Keywords:** preclinical, alcohol, drinking in the dark, behavioral flexibility, cytokine

## Abstract

The relationship between alcohol consumption and traumatic brain injury (TBI) often focuses on alcohol consumption increasing the likelihood of incurring a TBI, rather than alcohol use outcomes after TBI. However, patients without a history of an alcohol use disorder can also show increased problem drinking after single or multiple TBIs. Alcohol and mild TBI share diffuse deleterious neurological impacts and cognitive impairments; therefore, the purpose of these studies was to determine if an interaction on brain and behavior outcomes occurs when alcohol is consumed longitudinally after TBI. To examine the impact of mild repetitive TBI (rmTBI) on voluntary alcohol consumption, mice were subjected to four mild TBI or sham procedures over a 2 week period, then offered alcohol (20% *v*/*v*) for 2 weeks using the two-bottle choice, drinking in the dark protocol. Following the drinking period, mice were evaluated for neuroinflammatory cytokine response or tested for cognitive and behavioral deficits. Results indicate no difference in alcohol consumption or preference following rmTBI as compared to sham; however, increases in the neuroinflammatory cytokine response due to alcohol consumption and some mild cognitive behavioral deficits after rmTBI and alcohol consumption were observed. These data suggest that the cytokine response to alcohol drinking and rmTBI + alcohol drinking is not necessarily aggregate, but the combination does result in an exacerbation of cognitive behavioral outcomes.

## 1. Introduction

Alcohol use disorder (AUD) and traumatic brain injury (TBI) independently represent major health problems in the United States. Each year, over 14.5 million people are diagnosed with AUD [1] and more than 2.8 million people suffer a TBI that results in medical intervention [2,3]. However, these health concerns can have a comorbid presentation. The consumption of alcohol greatly increases the chances of sustaining a TBI, and as many as 50% of TBI emergency room patients have blood alcohol levels that surpass the legal limit [4,5]. Incurring a TBI also increases the likelihood of later developing an AUD independent of personal history of alcohol abuse [6,7,8,9,10]. Increased alcohol use following TBI is a substantial concern as it can greatly impact an individual’s recovery from TBI and lead to greater risk of incurring additional TBIs [11]. 

The majority of TBI cases, up to 90%, are concussions, also called closed head injuries, which are classified as “mild” [12,13]. Mild TBIs (mTBI) are identified by transient confusion, disorientation, or impaired consciousness, dysfunction of memory around the time of injury, and/or loss of consciousness lasting less than 30 min. Typically, it is difficult to observe tissue damage (primary injury) with standard MRI following mTBI [14,15]. However, there are a number of secondary mechanisms that occur in response to the primary injury that can also have damaging consequences [16,17]. Inflammatory responses are among the secondary effects that are a result of the upregulation of various proinflammatory cytokines and chemokines [18,19]. Early studies of neurological insult described the involvement of several cytokines and chemokines including interleukin-1β (IL-1β), IL-6, and tumor necrotic factor (TNF-α) [18]. The neurological symptom profile of chronic alcohol use has some similarities with mTBIs, specifically, various cognitive and behavioral deficits (i.e., memory deficits, confusion). These deficits are thought to be a result of alcohol’s neurotoxic properties that cause predictable neurodegeneration and an uptick in neuroinflammatory processes [18,20,21,22,23,24,25] including elevated proinflammatory cytokines, TNF-α, IL-1β, and IL-6 [26].

The intertwined presentation of mTBI and AUD, exacerbated recovery times for TBI after alcohol drinking, similar cognitive and behavioral deficits, and common neuroinflammatory processes suggest that these disorders may have shared neural mechanisms that can be targeted to reduce damage or improve cognitive and behavioral outcomes. These longitudinal experiments investigated the effects of mTBI on alcohol consumption using a well-characterized mouse model of repetitive mTBI (rmTBI) [27] and a well-established voluntary drinking model [27,28,29,30]. Furthermore, these studies sought to contribute to the growing body of literature examining the combined effects of rmTBI and alcohol drinking on the neuroinflammatory response via cytokine expression and cognitive behavioral outcomes.

## 2. Materials and Methods

### 2.1. Subjects

Sixty-four adult (8–12 weeks old) male C57BL/6J mice (Envigo Laboratories, Indianapolis, IN, USA) were used, with 12 mice per behavioral condition and four mice for the nonmanipulated RT-qPCR control. Food and water were freely available throughout the duration of the experiment, and all mice were maintained on a 12 h reverse light/dark cycle (6:00 p.m./6:00 a.m.) in a temperature- and humidity-controlled animal facility. Mice were transported to a separate room in the same building followed by a short habituation period (20 min) prior to rmTBI induction and behavioral assays, while all drinking measures occurred in the home cage. 

Following the final drinking session (24 h) or behavioral task (1 h), the mice were given a lethal dose of isoflurane, so that the brains could be cleared of blood via transcardial perfusion with phosphate-buffered solution and extracted for assessment of neuroinflammatory response.

These experiments were conducted according to a protocol approved by the Institutional Animal Care and Use Committee of the University of South Florida, in accordance with the National Institutes of Health Guide for the Care and Use of Laboratory Animals. IS00002500.

### 2.2. Repetitive Mild Traumatic Brain Injury

To model the rmTBI observed in humans, a conservative but validated model of closed head injury protocol was used [27]. Mice were anesthetized with 4% isoflurane and maintained at 2–3% isoflurane throughout the procedure. Using a PSI TBI-0310 Impactor (Precision systems and instrumentation, LLC, USA) fitted with a custom silicon rubber tip (1 cm in thickness, 9 mm in diameter), four brain traumas were induced at the center point of the closed skull with 72 h of recovery time between impacts. Each impact was made at 4.0 m/s, 3.8 mm compression depth, and a 200 ms dwell time (compression duration). Sham mice were anesthetized and placed in the impactor for a similar duration, but did not experience the impact. Mice were returned to home cages immediately after the injury procedure where they had access to ad libitum water and food and were monitored for health. After the four injuries or sham procedures, the mice would have an additional 4 days of recovery prior to alcohol exposure.

### 2.3. Voluntary Alcohol Consumption

**Figure 1:**
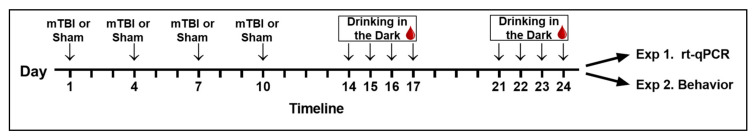
Timeline detailing the mild repetitive traumatic brain injury (rmTBI) or sham protocol and subsequent drinking in the dark (DID) schedule.

Voluntary alcohol consumption was determined using the well-established drinking in the dark (DID) protocol, two-bottle choice variant [28,29,30]. Alcohol solutions were made daily by diluting a 95% (*v*/*v*) ethyl alcohol (ethanol) with tap water to a 20% (*v*/*v*) concentration. Solutions were presented using sipper tubes featuring a ball bearing nozzle [29]. Alcohol was made available 3 h after the dark cycle had started and remained available for 2 h on Days 1–3 and 4 h on Day 4, whereas the mice remained abstinent on Days 5–7 (see Figure 1 for schematic of the timeline). All mice repeated this for two cycles of DID. During the alcohol intake period, mice were presented with a sipper filled with 20% alcohol solution and a second sipper of drinking water. Prior to placing the sippers on the cages and at the conclusion of the drinking period, the total volume of each solution (mL) was recorded. Alcohol preference was calculated for each session by dividing the alcohol volume (mL) consumed by the total (alcohol + water) volume (mL), and then converted to a percentage. The position of the bottles was alternated in a semi-random order each day to negate any side preferences. 

### 2.4. Blood Alcohol Concentration 

Blood samples were taken once per week immediately following the Day 4 drinking session via a standard submandibular bleed procedure. This procedure was very quickly conducted by an experienced researcher and did not require anesthesia. Samples were centrifuged (1500× *g*) following collection and the alcohol concentration measured using an Analox Instrument analyzer (Lunenburg, MA, USA). Plasma samples were analyzed immediately or stored at −20 °C until analyzed.

### 2.5. Experiment 1: Neuronal Cytokine Expression following rmTBI and Alcohol Drinking

#### 2.5.1. RNA Isolation

Frozen (whole) brains were brought to room temperature; TRIzol (Invitrogen, Carlsbad, CA, USA) was added and tissue was homogenized until no solid pieces were visible. Homogenized samples were incubated at room temperature for 5 min to allow for complete dissociation of the nucleoprotein complex. Chloroform was added to each sample, thoroughly mixed, and allowed to incubate another 2–3 min. Samples were centrifuged at 12,000× *g* for 15 min at 4 °C. The upper aqueous phase was removed and placed into a new collection tube. RNA purification occurred according to manufacturer protocol provided with PureLink RNA Mini Kit (Invitrogen, Carlsbad, CA, USA). An aliquot (5 µL) of RNA was diluted in 495 µL of DEPC-treated water in order to measure RNA concentration and purity. Each sample was assessed with ultraviolet (UV) spectroscopy utilizing the absorbance measured at 260 and 280 nm (GENESYS Spectrometer, Thermo Scientific). The remainder of the RNA sample was stored at −80 °C until further use.

#### 2.5.2. Complementary DNA (cDNA) Synthesis

The cDNA synthesis reaction was prepared according to manufacturer provided protocol included in the iScript Advanced cDNA Synthesis Kit for RT-qPCR (BioRad Laboratories, Hercules, CA, USA). Briefly, a working solution was made using the provided 5× iScript Advanced Reaction mix, iScript Reverse Transcriptase, and water. The sample RNA template was added to each well with the working solution. Samples went through reverse transcription by being held at 46 °C for 20 min and then 1 min at 95 °C to inactivate the reverse transcriptase. Samples were stored undiluted at −20 °C if not used for rt-PCR immediately. 

#### 2.5.3. Real-Time Polymerase Chain Reaction Assay

Quantitative PCR was conducted via manufacturer provided protocol for SsoAdvanced Universal SYBR Green Supermix (BioRad Laboratories, Hercules, CA, USA). Briefly, a working solution made using the provided SsoAdvanced Universal SYBR Green Supermix (2×), forward and reverse primers specific to the house gene (18S) or target gene (TNF-α, IL-1β, IL-6), and nuclease-free H_2_O. The samples were added to the plate in duplicate. Samples were then placed in iCycler MyIQ Real-Time PCR Detection System (BioRad Laboratories, Hercules, CA, USA) and heated to the protocol-specified temperatures for 35 cycles. 

Primer sequences used included TNF-α forward (F): 5’–GACCCTCACACTCAGATCATCTTCT, TNF-α reverse ®: 5’–CCTCCACTTGGTGGTTTGCT; IL-1β F: 5’–CTGGTGTGTGACGTTCCCATTA, IL-1β R: 5’–CCGACAGCACGAGGCTTT; IL-6 F: 5’–GGCCTTCCCTACTTCACAAG, IL-6 R: 5’–ATTTCCACGATTTCCCAGAG. 

### 2.6. Experiment 2: Cognitive Behavioral Performance following rmTBI and Alcohol Drinking 

Cognitive and behavioral tasks included the Serial Spatial Discrimination Reversal Learning (SSDRL) task measuring simple discrimination and behavioral flexibility and an integrated open-field (OF) task measuring spontaneous locomotor activity and novelty preference; see Table 1 for testing schedule. The SSDRL task is a general measure of global cognitive function and behavioral flexibility. It required mice to learn a strategy using spatial cues, recognize when this strategy no longer works, and switch to a new strategy. The OF test provided a measure of general locomotor activity after habituating to a new environment and further trials assessed novelty preference. 

#### 2.6.1. Serial Spatial Discrimination Reversal Learning

The SSDRL task uses a partially submerged T-maze (overall upper arm length 24 in, arm width 3–3.5 in, base leg length 15 in, wall height 15 in), in which mice were required to learn to escape from the water (24–26 °C, dyed opaque with nontoxic, water-soluble white paint) using a platform at one of the arms [31,32,33,34]. on the first day of this task, there was a pretrial training session to habituate the mice to the task. The arms of the T-maze were blocked in a pseudorandom order to aid mice in learning to escape from both sides of the maze at least five times prior to testing. During the subsequent 3 days of testing, mice learned to escape by remembering the location of the escape platform on either the left or right arm of the maze. Once a rule was sufficiently learned (criterion of six correct choices with no errors), the rule was reversed so that the platform was on the opposite side of the maze. Mice completed 30 trials per day and were kept in a warmed environment (approximately 31 °C) between trials (1–2 intertrial minute intervals) to prevent hypothermia.

All mice started testing on their nonpreferred arm. This was determined by allowing the animal to choose freely between the two arms on the first test trial and then the escape platform was placed in the opposite arm. Errors included entering the incorrect arm, re-entry to the starting stem, and backtracking through the correct arm without escaping. Because the criterion to reverse the contingencies was six correct trials, if an animal completed two consecutive trials during the final trials of the day, an additional four trials were added in an attempt to allow the animal to reach the criterion. If the mouse made an error during one of the extra trials, testing ended for the day. 

#### 2.6.2. Open Field with Novelty Preference Task

These tasks assessed locomotor activity (16.75 in × 16.75 in; distance traveled), preference for novelty, and working memory (via recognition of familiar object and time spent with novel object). On Day 5, mice freely explored the arena for 3 min to habituate. Approximately 30 min later, an additional 3 min session took place to assess locomotor activity. On Day 6, a 3 min exploration included a novel object. After a 30 min delay, a second object was added to the arena and the mouse was allowed another 3 min to explore. The objects were solid-colored, hard plastic geometric toys (sphere, cube, and pyramid) approximately 1.25 in tall. The objects were epoxied to a thin ceramic tile, ensuring the mouse could interact with but not move the toy. The arena and all objects were cleaned with 40% ethanol wipes to sanitize and deodorize between mice. 

### 2.7. Analyses

All analyses were conducted with Prism (Version 9, GraphPad Software, San Diego, CA, USA). A *p*-value less than 0.05 was set to evaluate significance unless otherwise indicated. Outliers were identified and removed following the use of Grubbs’ test for a single outlier. Where appropriate, an unpaired *t*-test or a one-way ANOVA with corrected multiple comparisons (Holm–Šídák) was used to compare between groups. Blood alcohol content (BAC) was analyzed with a simple correlation. Means are reported ± the standard error of the mean (SEM). 

Relative quantification of gene expression of cytokines (TNF-α, IL-1Β, and IL-6) was calculated as fold change using the 2^−ΔΔCT^ method comparing changes in house gene (18S) to target gene in controls and experimental groups [35]. IL-1Β and IL-6 expression was examined in follow-up to the TNF-α assay. To conserve the sample, IL-1Β, IL-6, and the house keeping gene 18S were assessed on a single 96-well plate with samples run in duplicate. Samples were excluded from analysis if the difference in Ct between duplicates was greater than 0.5 Ct. 

## 3. Results

### 3.1. Experiment 1: rmTBI on Voluntary EtOH Intake and Combined Effects on Neurocytokine Expression

#### 3.1.1. Voluntary Alcohol Consumption

**Figure 2:**
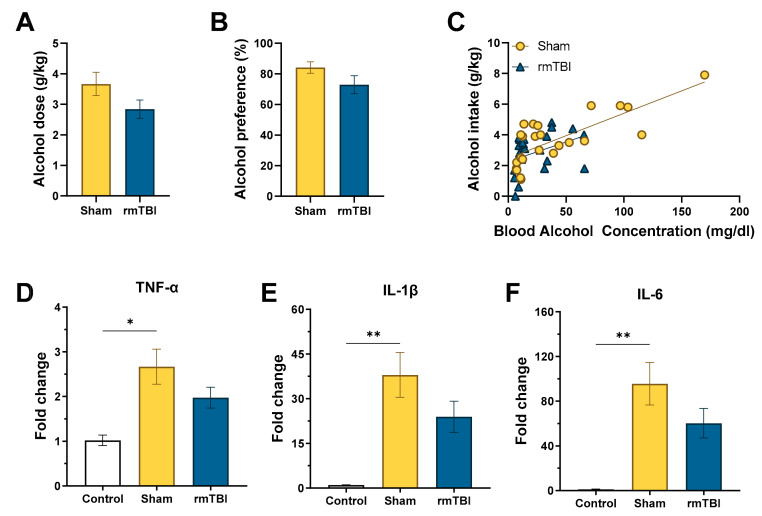
Alcohol drinking resulted in increased cytokine expression: (**A**) alcohol intake and (**B**) preference (*n* = 12/group), did not differ as a result of rmTBI (**C**) and BAC (mg/dL), (*n* = 24/group); (**D**) tumor necrosis factor (TNF)-α (control *n* = 4, sham *n* = 10, rmTBI n=9), (**E**) interleukin (IL)-1β (control *n* = 3, sham *n* = 5, rmTBI *n* = 6), (**F**) and IL-6 (control *n* = 3, sham *n* = 5, rmTBI *n* = 6) all showed pronounced increase of expression following 2 weeks of alcohol consumption.

As shown in Figure 2, the average alcohol intake and preference for the 4 h drinking sessions at the end of Week 1 and 2 were calculated for interpretation of the DID, two-bottle choice voluntary drinking protocol. No differences were observed in intake between sham (*M* = 3.67 g/kg ± 0.38) and rmTBI (*M* = 2.84 g/kg ± 0.30) groups, *t*(22) = 1.70, *p* = 0.052 or alcohol preference Sham (*M* = 84.17% ± 3.717%) and rmTBI (*M* = 2.92% ± 5.89%), *t*(22) = 1.62, *p* = 0.060. However, these results suggest that there could be a small decrease in alcohol drinking after rmTBI, but this difference was not replicated in the intake measures of the second experiment, and the effect size (*R* = 0.116) was minimal; therefore, it was not considered a meaningful trend. Results of the BAC analysis showed a positive Pearson’s correlation for both sham (*r* = 0.76, *p* < 0.001) and rmTBI (*r* = 0.39, *p* = 0.004) groups.Comparison of the sham (*M* = 41.15 mg/dL) and rmTBI (*M* = 22.22 mg/dL) average BAC did reveal a difference between groups, *t*(46) = 1.99, *p* = 0.026, indicating differing levels of intoxication following the 4 h drinking period. The results of the voluntary alcohol intake (g/kg), alcohol preference, and BACs fell within expected range for the DID, two-bottle choice protocol [28,36,37,38,39]. Ultimately, these results provide support for the use of this drinking model to elicit voluntary alcohol consumption and high alcohol preference following the sham or rmTBI procedure.

#### 3.1.2. RT-qPCR

Alcohol drinking increased cytokine expression for TNF-α, IL-1β, and IL-6. TNF-α expression more than doubled following 2 weeks of alcohol drinking, *F*(2, 20) = 4.50, *p* = 0.025, for control (No alcohol + no rmTBI; *M* = 1.02 ± 0.12), sham + alcohol drinking (*M* = 2.67 ± 0.39), and rmTBI + alcohol drinking (*M* = 1.98 ± 0.24). Holm–Šídák’s multiple comparisons test confirmed a statistical difference between control and sham + alcohol drinking (adjusted *p* = 0.024); however, the increase observed after rmTBI + alcohol drinking did not meet the statistical threshold after multiple correction comparisons to control or sham + alcohol drinking groups (adjusted *p* = 0.206 for both comparisons). 

An additional evaluation of IL-1β and IL-6 expression showed similar patterns to TNF-α, but with notably higher fold changes following sham + alcohol drinking (IL-1β, *F*(2,11) = 7.17, *p* = 0.010). As shown in Figure 2, IL-1β expression showed a nearly 40-fold increase, control (*M* = 1.01 ± 0.09), sham (*M* = 37.97 ± 7.54), which was statistically significant after Holm–Šídák’s multiple comparisons test (adjusted *p* = 0.009). Surprisingly, the rmTBI + alcohol drinking group did not reach the statistical threshold despite a more than 20-fold increase compared to control (*M* = 23.93 ± 5.25; adjusted *p* = 0.066) or to the sham + alcohol drinking group (adjusted *p* = 0.111). There was an even greater increase in IL-6 expression, *F*(2, 11) = 7.39, *p* = 0.009, after sham + alcohol drinking (*M* = 95.62 ± 18.98) compared to control (*M* = 1.12 ± 0.32) (adjusted *p* = 0.008). Again, the rmTBI + alcohol drinking group (*M* = 60.28 ± 13.23) did not meet the statistical threshold in adjusted comparisons to control (adjusted *p* = 0.059) or sham + alcohol drinking (adjusted *p* = 0.111). The large differences but lack of statistical significance for the IL-1β and IL-6 multiple comparisons are likely attributable to the reduction in sample size and, therefore power, in an effort to conserve the sample and compare both targets on a single 96-well plate. However, the effect size (IL-1β, *R*^2^ = 0.573; IL-6, *R*^2^ = 0.566) was large enough to lend confidence that the results are meaningful and worthy of further exploration in future studies. 

### 3.2. Experiment 2: rmTBI on Voluntary EtOH Intake and Combined Effects on Cognitive Behavioral Performance

#### 3.2.1. Voluntary Alcohol Consumption

**Figure 3:**
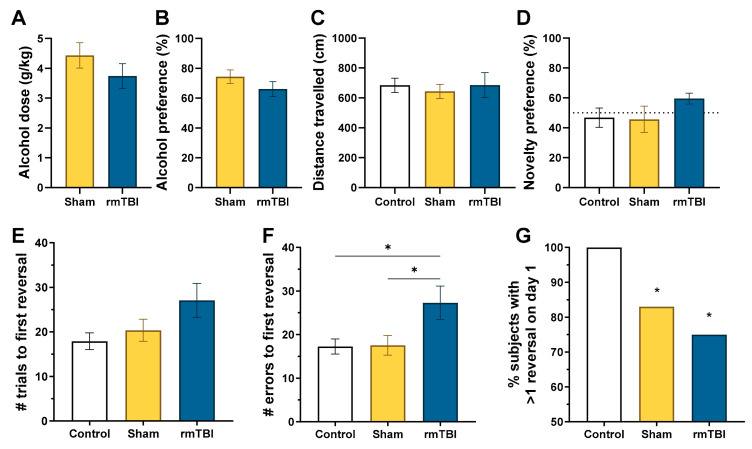
rmTBI + alcohol drinking resulted in mild behavioral flexibility deficits: (**A**) alcohol intake and (**B**) preference (*n* = 12/group); (**C**) no differences in locomotor activity or (**D**) novelty preference were observed (control *n* = 12, sham n=12, rmTBI *n* = 11). (**E**) The number of trials required to reach first reversal; (**F**) the number of errors committed prior to first reversal are a more sensitive measure of deficits in behavioral flexibility; (**G**) the number of mice able to achieve at least one reversal in the first training day was decreased by alcohol drinking and rmTBI + alcohol drinking (control *n* = 11, sham *n* = 11, rmTBI *n* = 11); * significantly different from control group (exact binomial test).

As shown in Figure 3, the voluntary alcohol drinking findings were replicated in Experiment 2, showing no differences in the average alcohol intake between sham (*M* = 4.43 g/kg ± 0.42) and rmTBI (*M* = 3.74 g/kg ± 0.41) groups, *t*(22) = 1.059, *p* = 0.129, or in alcohol preference between sham (*M* = 74.42% ± 4.522%) and rmTBI (*M* = 66.08% ± 5.01%), *t*(22) = 1.226, *p* = 0.115. 

#### 3.2.2. Cognitive Behavioral Assessment

During the battery of cognitive and behavioral tasks, locomotor activity (total distance traveled) was similar for all three groups, *F*(2, 32) = 0.87, *p* = 0.431, control (*M* = 48.50 ± 6.51), sham + alcohol drinking (*M* = 38.50 ± 5.53), and rmTBI + alcohol drinking (*M* = 48.73 ± 6.93). Similarly, no differences were observed in the novelty preference task for percent time spent with the novel object, *F*(3, 42) = 1.16, *p* = 0.077, control (*M* = 46.75% ± 6.45%), sham + alcohol drinking (*M* = 45.64% ± 8.80%), and rmTBI + alcohol drinking (*M* = 59.50% ± 3.64%). 

The SSDRL task revealed decreased cognitive behavioral performance for the alcohol drinking and rmTBI + alcohol drinking groups. For the rmTBI + alcohol group, the number of trials needed to reach the first reversal was slightly increased but did not statistically differ, *F*(2, 30) = 2.81, *p* = 0.076, control (*M* = 17.91 ± 1.87), sham + alcohol drinking (*M* = 20.36 ± 2.48), and rmTBI (*M* = 27.09 ± 3.81). However, the cognitive deficits became readily apparent in the number of errors made through the first reversal, *F*(2, 30) = 4.231, *p* = 0.024. The number of errors for the control (*M* = 17.27 ± 1.73) and sham + alcohol drinking (*M* = 17.55 ± 2.26) groups was similar, but the rmTBI + alcohol drinking group (*M* = 27.27 ± 3.86) showed an increase compared to the other two groups (adjusted *p* = 0.047 in both corrected comparisons). Finally, to compare the percentage of mice able to achieve at least one reversal on the first training day, an exact binomial test was conducted for each group with the expected outcome of 100% success. All control mice were able to achieve at least one reversal on the first training day, in contrast to 83% of sham + alcohol drinking mice (*p* < 0.001), and 75% of rmTBI +alcohol drinking mice (*p* < 0.001). 

## 4. Discussion

In both experiments, the DID two-bottle choice protocol produced consistent alcohol intake. However, there was no evidence for elevated voluntary alcohol consumption or an escalated drinking pattern following this model of rmTBI as compared to sham. In fact, the data suggested that there might be differing intake patterns over the 4 h drinking period, resulting in different end-point BAC levels. In our study, the sham mice had higher BAC levels at the end of the 4 h drinking period, and only sham mice reached intoxicating BAC levels at the collection point. While these data are very enticing and suggest differing intake patterns after rmTBI, perhaps due to differing subjective effects of alcohol after injury, this would need to be investigated via time course intake and BAC measures in future studies. Results from the current study are in contrast to other studies that showed bimodal changes in alcohol drinking or elevated drinking after TBI [40,41]. There are notable differences in the mechanism of TBI, the voluntary alcohol protocol employed, and rodent models used in both studies as compared to the current experiments. For example, the current study used a closed head model with a rubber-tipped piston without exposing the skull. This in contrast to the blast-induced TBI model and the acute closed head injury model with a metal piston directly impacting the skull. These different methods all result in mild injury similarly characterized as without skull fracture or hemorrhage; hence, it is possible, but unlikely, that variations in injury induction drive alcohol drinking differences. 

Alternatively, rodent strains have different voluntary alcohol consumption patterns that could account for the different outcomes. Sprague-Dawley rats are generally considered a low-alcohol preferring model, whereas the C57BL/6J mice used in the current study normally drink high volumes of alcohol without training or forced consumption [39,42,43,44,45]. The Swiss-Webster outbred mice that showed elevated adult alcohol consumption also tend to drink less than the C57BL/6J strain. Furthermore, the voluntary consumption protocols have different strengths and limitations [28,30,46]. The objective of the DID protocol is to produce high levels of alcohol consumption in a short amount of time, characterizing it as a binge model. Given that the chosen mouse strain, C57BL/6J, is considered a high-alcohol preferring strain, this may have inadvertently led to a ceiling effect when used in the DID protocol in the current study. Together, our intake and BAC results also indicated there may be time course differences in alcohol consumption after rmTBI, which would need to be explored in future studies. Our results showed a strong preference for alcohol for both the sham and the rmTBI groups, which could be indicative of a ceiling effect on alcohol consumption during the limited intake window. Finally, given the altered drinking in studies using outbred rodent strains, it is possible that the genetically homogenous C57BL/6J mouse strain is not able to model altered alcohol consumption after rmTBI. Even in clinical populations, only a portion of patients are reported to excessively drink following TBI without previous history of AUD, which suggests that specific genetic factors may play an important role in divergent outcomes in rodent models of alcohol consumption following TBI [4,47,48]. 

It was expected that alcohol drinking would elevate neuroinflammatory markers in the current study. This hypothesis was supported by the observed elevated expression of TNF-α, IL-1Β, and IL-6 cytokines in the sham + alcohol drinking group. It was also predicted that the combination of rmTBI and alcohol consumption would together compound the independent increase in cytokine expression observed after alcohol exposure and in TBI models. However, despite the rmTBI group consuming similar amounts of alcohol, there was not a significant increase in expression for any of cytokines probed, suggesting a more complicated cytokine response from the combined neuronal insult of alcohol drinking and rmTBI. 

The recruitment of cytokines can be incredibly complex and occurs via the Toll-like receptor (TLR) or the receptor for advanced glycation end products (RAGE) pathways, each with unique intracellular mechanisms that lead to altered gene expression of cytokines [49]. TNF-α and IL1-β are proinflammatory and considered neurotoxic; they exacerbate TBI and extend recovery times, initiate inflammatory processes, disrupt the BBB, and recruit leukocytes [50,51]. Alternatively, IL-6 and IL-10 are generally considered neuroprotective as evidence suggests that IL-6 can reduce apoptotic cell death and promote neuroregenerative factors, and IL-10 can reduce TNF-α induced inflammation [52,53]. IL-6 and IL-1 have also been shown to be significantly upregulated in patients with the most severe and even fatal TBI, as they are presumably recruited to sites of injury in response to tissue damage [54,55].

In acute doses, alcohol has been reported to suppress some of the TLRs, thereby attenuating proinflammatory cytokines [56,57,58]. However, chronic alcohol exposure is more commonly associated with increases in proinflammatory cytokines (IL-1β, IL-6, and TNF-α) [59,60,61]. Likewise, studies have shown an increased proinflammatory cytokine response when alcohol was present at the time of injury [62] or following injury [19]. Studies observing cytokine protein response following TBI in rodents also observed increases in these same proinflammatory cytokines [18,51,63], and a recent study in humans showed an acute elevation of IL-1β and IL-6 in blood serum up to 1 month after mTBI [64]. In many of the published studies, cytokine assessment was done via ELISA (measuring protein expression), while others used RT-PCR (assessing gene expression). Gene expression changes are not necessarily followed by similar magnitude changes in protein expression; thus, it can be difficult to make direct comparisons across the literature. Our experiments assessed gene expression and showed large magnitude differences after sham + alcohol drinking and increased, but not statistically different, expression in rmTBI + alcohol drinking. Future studies will be required to understand if these gene expression changes would result in similar protein changes. Despite different methods of TBI induction and alcohol exposure across the literature, it is encouraging to see similar directional changes in cytokine gene and protein expression following neural insult. 

To further complicate assessing the response to neural insult via alcohol and/or injury, the recruitment of cytokines can independently occur at neurons, microglia, and astrocytes, each of which may be mediated by different factors [49,62]. It should also be noted that these studies measured cytokine gene expression in whole brains, because the injury was global instead of focal. Nevertheless, further investigation into brain region-specific response to injury is important to understand the cognitive deficits observed in the second experiment. Future studies should consider measuring multiple cytokines simultaneously (i.e., multiplex array of gene and/or protein expression), taking histological measures of microglia or astrocyte reactivity and region-specific inflammatory responses, and examining upstream targets such as HMGB1, TRL, and RAGE. This additional information would provide a more complete understanding of the cytokine cascade response to alcohol drinking after rmTBI. 

The results of our cognitive behavioral battery supported the hypothesis that rmTBI + alcohol drinking together would lead to worse cognitive and behavioral outcomes compared to alcohol drinking alone. This was most evident in the decreased percentage of subjects in the sham + alcohol drinking group that were able to achieve a reversal of the simple discrimination rule in the first training day, with a further decrease for the rmTBI + alcohol subjects. Looking more closely at the data, the rmTBI + alcohol drinking group was able to achieve reversal with only slightly more trials, but these mice committed more errors compared to the sham + alcohol drinking and control groups. This suggests that the rmTBI + alcohol drinking mice had mild cognitive deficits that resulted in loss of behavioral flexibility and increased perseverative errors. Our data did not show any changes in locomotor activity or novelty preference, which suggests the observed impairments are not due to motor difficulties or basic short-term memory deficits. 

While animal models of repetitive mTBI do often demonstrate poor cognitive and behavioral outcomes after multiple mTBIs [27,65,66], previous studies reported mixed results on cognitive performance when alcohol was administered prior to or following acute and rmTBI in rodent models. For example, one study found no effects on neurological or behavioral outcomes when alcohol was administered prior to acute mTBI [62], and others showed delayed recovery of sensorimotor function [67]. Others found that alcohol administered chronically after mTBI produced mild neurological deficits, impaired memory on a novel object recognition task, and decreased locomotor activity [68,69]. The inability of models to produce consistent cognitive deficits from mTBI and alcohol exposure impresses upon the importance for continued research [62,69]. Furthermore, our primary research question focused on the impact of alcohol drinking or rmTBI + alcohol drinking; however, it is also important to consider how rmTBI + alcohol drinking differs from rmTBI alone. While the scope of the current studies is limited, the literature would greatly benefit from future studies able to make both comparisons.

As previously discussed, any alteration in alcohol administration, timing or severity of injury, or rodent strain could explain the incongruent outcomes reported here and throughout the literature. For instance, it is possible that the doses of alcohol consumed in this study were not sufficient to elicit more dramatic alcohol-induced deficits in behavioral flexibility that have been previously observed [31,32,33,34,70]. Likewise, concussive deficits produced by rmTBI are often transient in nature with symptoms appearing and disappearing over extended periods, and the injuries globally impact the brain. For example, some researchers have shown mTBI deficits that were only apparent 30 days after injury [71]. The mild injury and the levels of alcohol consumed in this protocol lend to a highly translational model of rmTBI and binge-like alcohol drinking, but they are also likely responsible for less robust cognitive and behavioral changes. 

Future studies should consider the administration of alcohol at low, moderate, and high doses following rmTBI to better control alcohol-mediated effects. Researchers using animal models could also use highly innovative measures such as diffusion tensor imaging (DTI) imaging to assess and correlate the integrity of white-matter tracts after rmTBI to executive functions such as reversal learning and working memory. Lastly, this study only addressed cognition and behavior post injury in alcohol-naïve mice, whereas, in humans, it is more likely that alcohol exposure starts prior to injury. Future studies should consider pretreatment of alcohol and possibly even the use of alcohol-dependent mice with the rmTBI model to assess changes in cognition and behavior. 

## 5. Conclusions

Our results show a deleterious impact of alcohol drinking on neural cytokine response, but suggest a more complex interaction when rmTBI and alcohol drinking were combined. Further, our data demonstrate mild behavioral flexibility deficits and increased perseverative errors in mice after rmTBI + alcohol drinking compared to alcohol drinking alone. Together, the current studies highlight the importance and complexity of investigating comorbid presentation of rmTBI and alcohol drinking.

## Figures and Tables

**Table 1:** Cognitive behavioral assessment schedule. SSDRL, Serial Spatial Discrimination Reversal Learning.

Cognitive Behavioral Battery Schedule	
Day 1	SSDRL: pretrial training (10 trials)
Day 2	SSDRL: test day (30 trials)
Day 3	SSDRL: test day (30 trials)
Day 4	SSDRL: test day (30 trials)
Day 5	Open field: habituation, (trial 1); 30 min delay; locomotor, (trial 2).
Day 6	Novelty preference: open field + novel object (trial 3); 30 min delay; familiar object + novel object (trial 4).
